# Supplementary material for: Ciliary exclusion of Polycystin-2 promotes kidney cystogenesis in an autosomal dominant polycystic kidney disease model
Source: Nat Commun. 2019 Sep 6;10:4072. doi: 10.1038/s41467-019-12067-y (PMC6731238; doi:10.1038/s41467-019-12067-y)
Supplement: Supplementary file 1 — Supplementary Information [file 41467_2019_12067_MOESM1_ESM.pdf]

Ciliary exclusion of Polycystin-2 promotes kidney cystogenesis in an Autosomal Dominant Polycystic Kidney Disease model

Walker et al

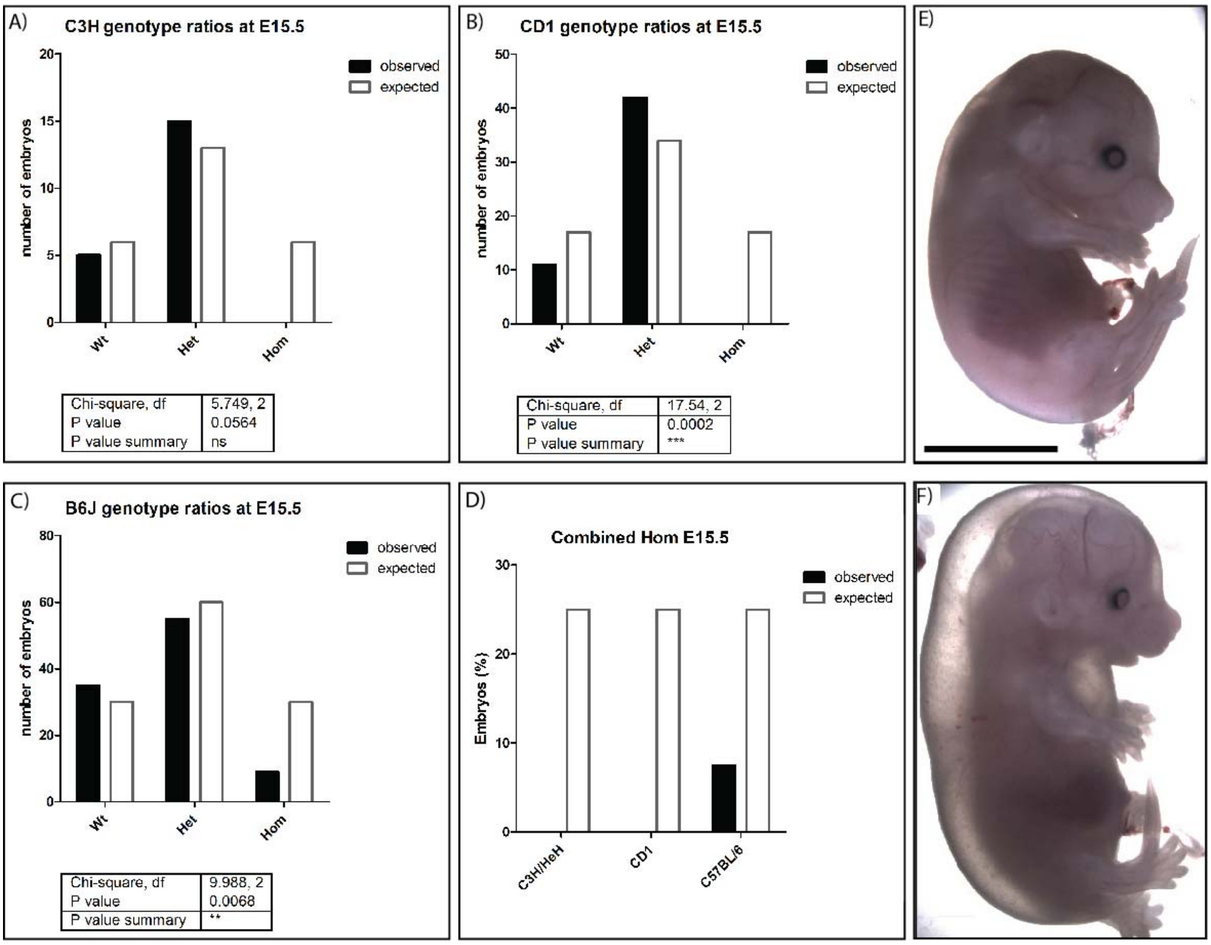

Supplementary Figure 1: Changing genetic background increased longevity

Survival of *Pkd2*$^{lrm4/lrm4}$embryos on different genetic backgrounds at E15.5. A) *Pkd2*$^{lrm4/lrm4}$-C3H embryos do not survive to E15.5. B) On a more outbred background, *Pkd2*$^{lrm4/lrm4}$-CD1 mice did not survive to E15.5. C) Of the strains examined, only *Pkd2*$^{lrm4/lrm4}$-C57BL/6J survived to E15.5. D) Only the C57BL/6J genetic background was permissive to homozygote survival at E15.5. E) Pkd2$^{+/+}$-C57BL/6J embryo at E15.5 develops normally. Scale bar 1mm. F) Pkd2$^{lrm4/lrm4}$-C57BL/6J embryos at E15.5 develop visible oedema.

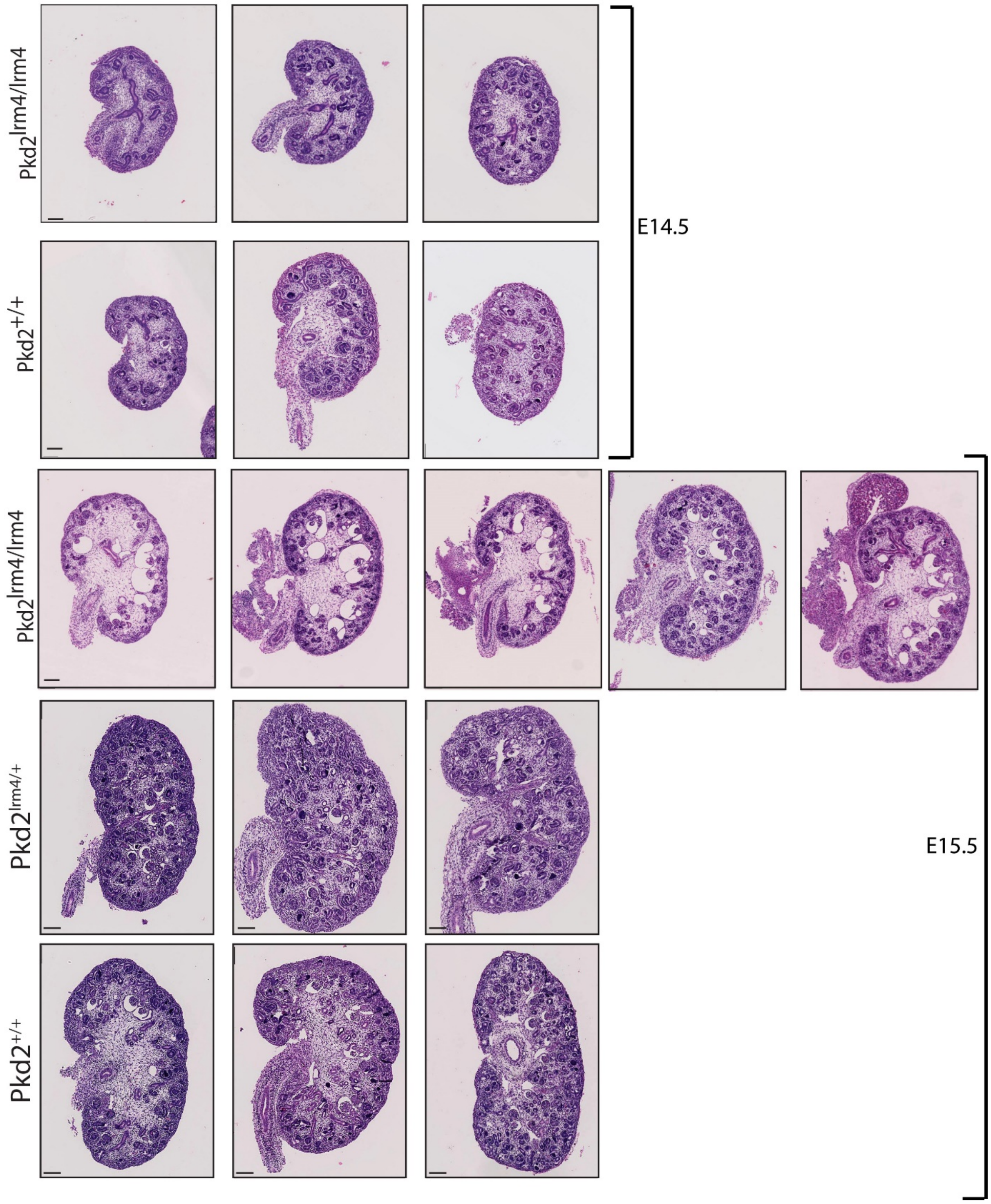

Supplementary Figure 2: Embryonic Kidneys at E14.5-E15.5

Images of E14.5 and E15.5 kidneys for comparison of phenotypic variation. At E14.5 homozygotes and Wt kidneys are indistinguishable. No cysts are observed. At E15.5, only *Pkd2*$^{lrm4/lrm4}$ kidneys exhibit cysts. Each image is representative on one kidney from an individual embryo. Scale bar 100um.

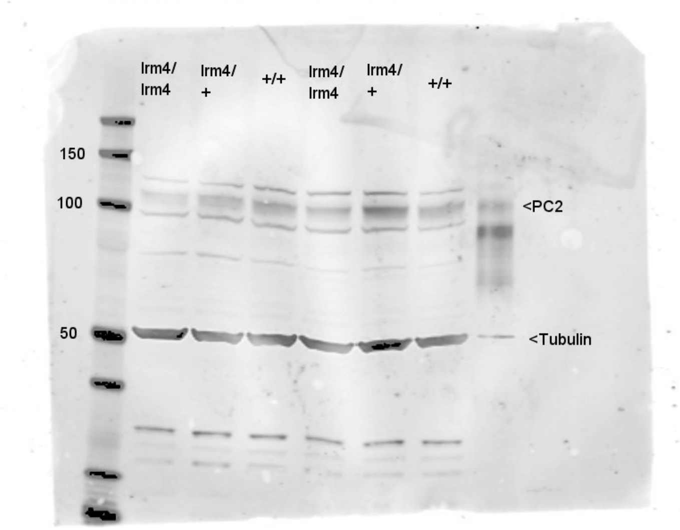

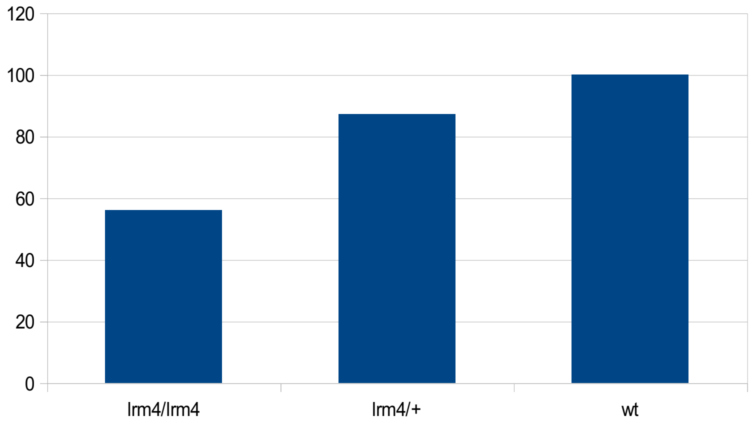

Supplementary Figure 3: Western blot of PKD2 protein in MEF lysate

Western blot of PC2 (at ~110KDa) from primary MEF showing reduced protein levels in PKD2$^{lrm4/lrm4}$ compared to $^{+/+}$ or $^{lrm4/+}$ samples, when normalised to tubulin (at 50KDa) loading control. Graph shows densitometric analysis of bands normalised to tubulin (Y axis is % of Wt protein). Pkd2$^{lrm4/lrm4}$ is reduced to 56.08% of the wt level.

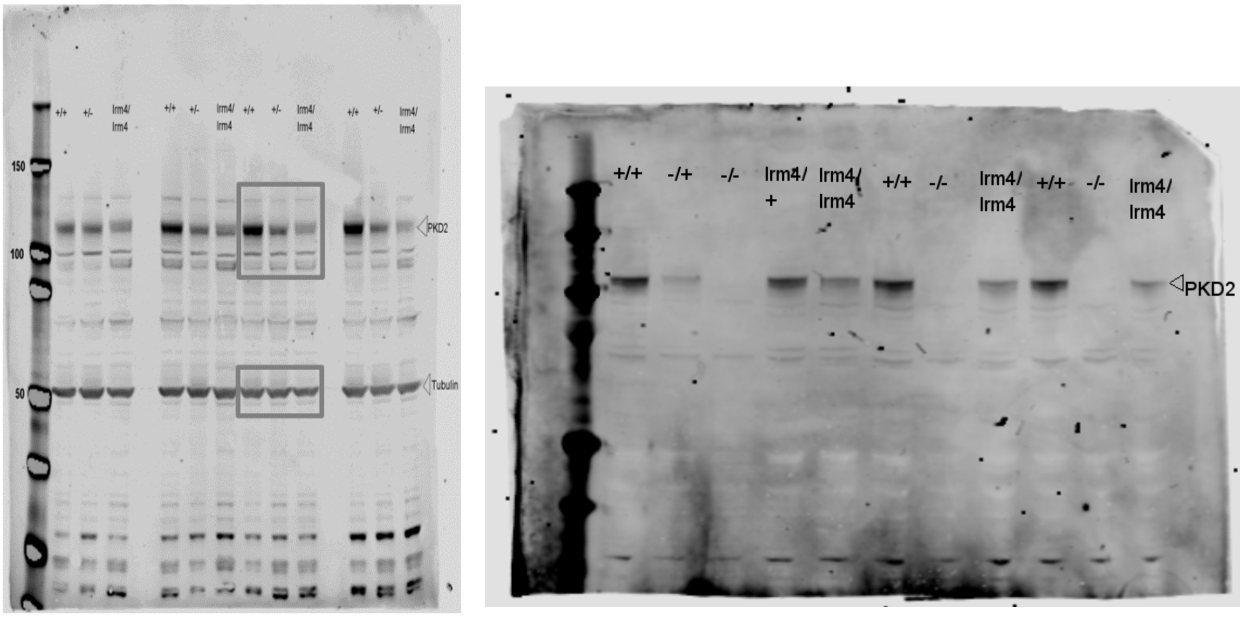

Supplementary Figure 4: **Full membrane from Fig2a showing embryonic kidney lysates from** $\boldsymbol{Pkd2^{lrm4/lrm4,}}$ $\boldsymbol{Pkd2^{+/-}}$**,** $\boldsymbol{Pkd2^{+/+}}$ **samples.**

Gel loaded with 4 biological repeats for each sample. Each sample constitutes a pool of 3 pairs of E14.5 embryonic kidneys. Western blot shows that PKD2 protein level is similar in $Pkd2^{lrm4/lrm4}$ and $Pkd2^{+/-}$ samples but that both are reduced compared to those of $Pkd2^{+/+}$samples. Box indicates region used for Fig2A

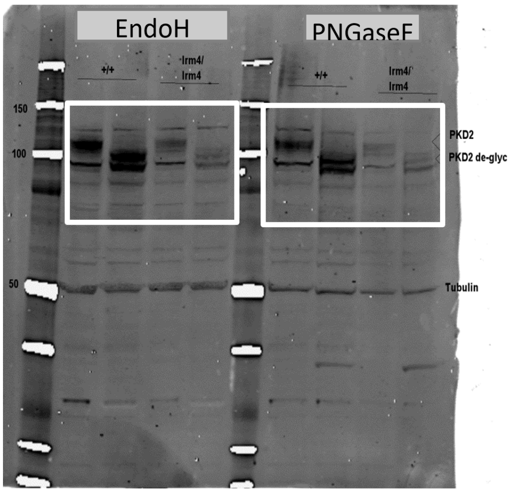

Supplementary Figure 5: Full membrane from Fig 2E showing glycosylation assay

Western blot of whole cell lysate from *Pkd2*$^{+/+}$ and *Pkd2*$^{lrm4/lrm4}$ MEF cells. Digestion with PNGaseF and EndoH produce faster running fractions in both samples. Boxes show regions used for Fig2E

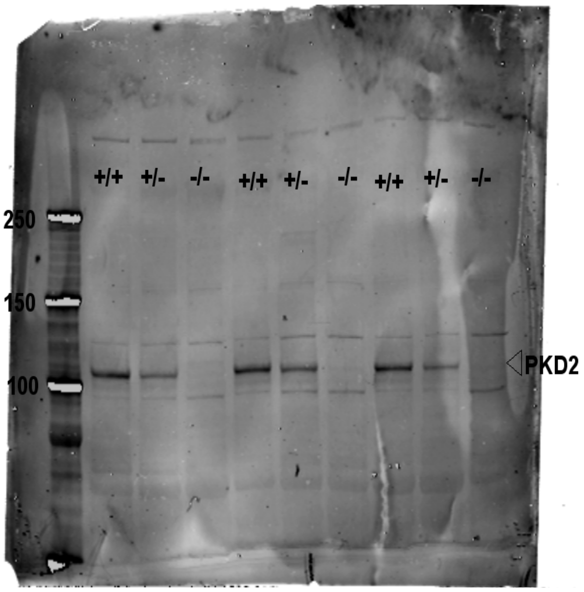

Supplementary Figure 6: Western blot of *Pkd2*$^{-/-}$ MEFs, revealing non-specific bands

(Garcia-Gonzalez et al., 2010)[1] show that PKD2^{-} cannot be recognised by either C- or N-terminal antibodies. Here, H-280 (SC-25749) an antibody recognising the C-terminal portion of the protein is used. On WB, no PKD2 protein is detected in the Pkd2$^{-/-}$ samples. A number of non-specific bands can thus be identified when detecting the Wt protein.

A) Mouse PKD2 glycosylation graph:

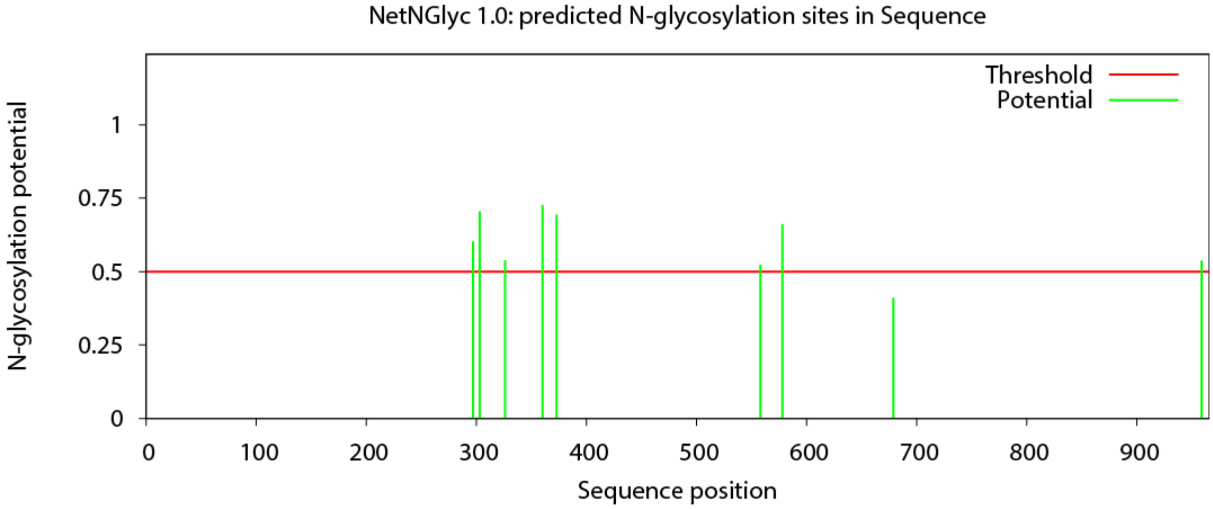

B) Human PKD2 glycosylation graph:

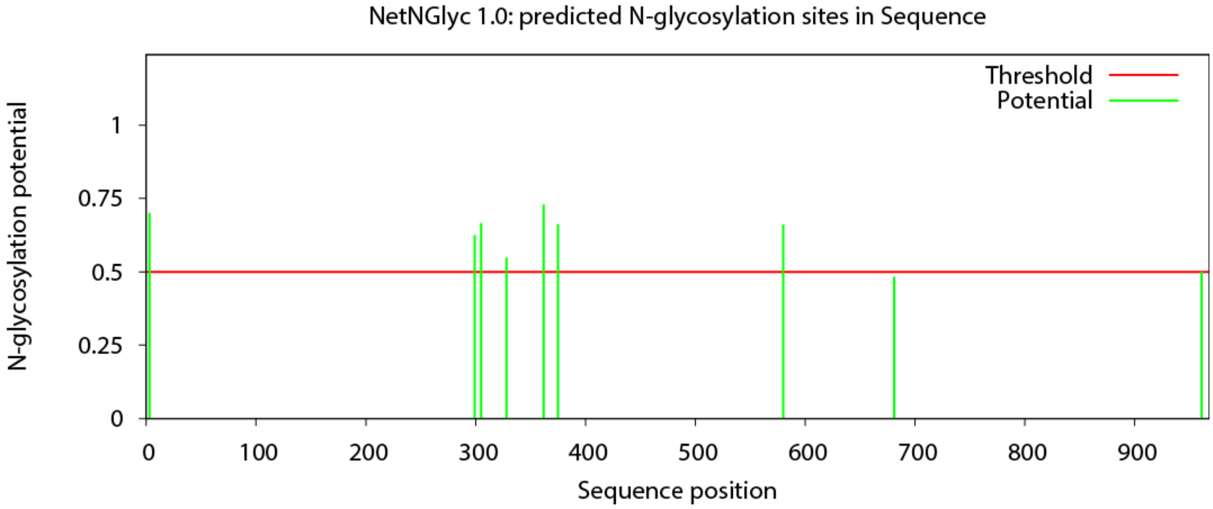

Supplementary Figure 7: Glycosylation Graphs

A) PKD2 mouse sequence N-glycosylation map. B) PKD2 human sequence N-glycosylation map. N-glycosylation sites (green lines) were predicted using the NetNGlyc (http://www.cbs.dtu.dk/services/NetNGlyc/) online tool. Nine glycosylation sites were predicted for both human and mouse PKD2 sequence. The diagram in Fig 2.D is comprised from the two graphs above.

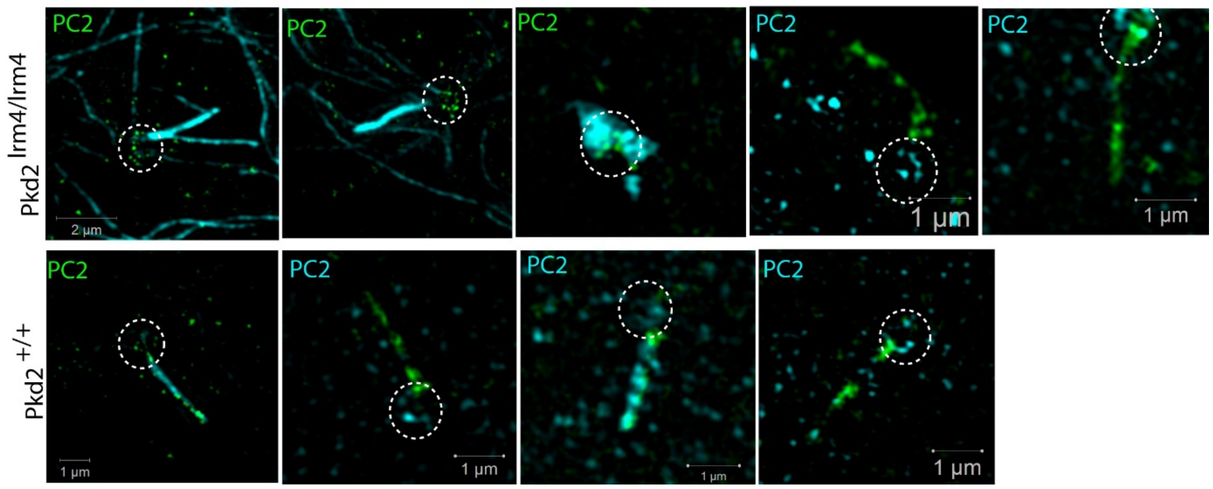

Supplementary Figure 8: comparison of PC2 ring at the base of Wt and Pkd2$^{lrm4/lrm4}$ MEF cilia in SIM images

A 1um dia circle was drawn at the base of the cilium to compare intensity of the ring of PC2 in Wt and Pkd2$^{lrm4/lrm4}$ images. The intensity of the grey values within a 1um dia circle at the base of the cilium was measured in the PC2 channel of each image. Although the mutant rings were on average more intense (13.08 vs 10.74 mean intensity value), this was not a significant trend.

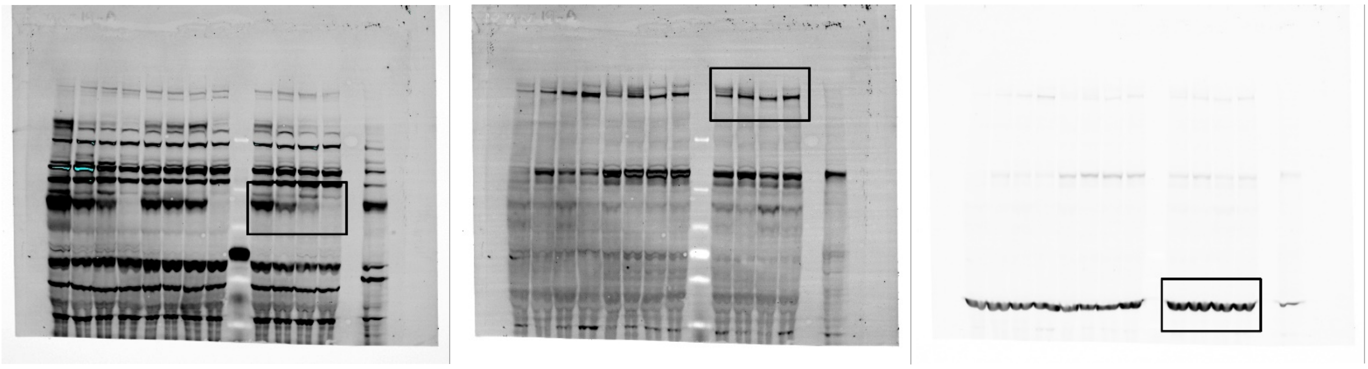

Supplementary Figure 9: PC1 levels are not reduced in *$PC2^{lrm4/lrm4}$* MEF cells

Western Blot analysis of PC1 expression in PC2 cells reveals that there is no overall reduction in PC1 in *$Pkd2^{lrm4/lrm4}$* cells. Single channel images from single blot; 1st image: PC2 Blot (H280 anti PC2), 2nd image: PC1 blot (7e12 anti PC1), 3rd image:β-actin loading control. Lanes (loaded from left to right) 1: *$Pkd2^{+/+}$*, 2: *$Pkd2^{+/-}$, 3: $Pkd2^{lrm4/lrm4}$*, 4: *$Pkd2^{-/-}$*, 5: *$Pkd2^{+/+}$*, 6: *$Pkd2^{+/-}$, 7: $Pkd2^{lrm4/lrm4}$*, 8: *$Pkd2^{-/-}$, 9: ladder, 10: $Pkd2^{+/+}$, 11: $Pkd2^{+/-}$, 12: $Pkd2^{lrm4/lrm4}$13: $Pkd2^{-/-}$, 14: empty, 15:transfected PC1 and PC2 control HEK*

Full images of blot from Fig 4

A) B)

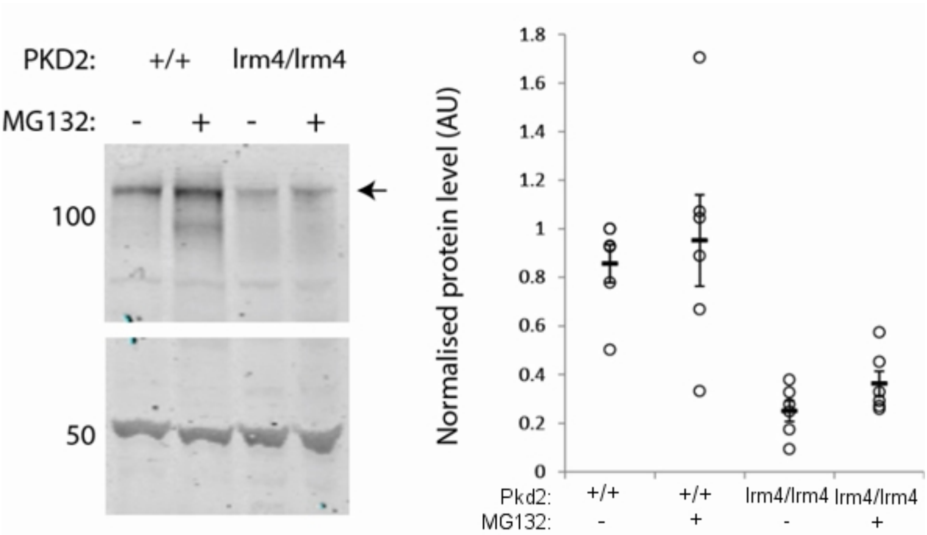

Supplementary Figure 10: Proteasomal degradation accounts for a small proportion of PC2 protein reduction in *Pkd2*$^{lrm4/lrm4}$

A) Western blot of protein from cells treated with proteasome inhibitor, MG132, for 10 hours. Blot probed with PC2 antibody (arrow at ~110KDa). Band at 50KDa depicts tubulin loading control. B) The graph represents the intensity of each lane from two experiments of 3 biological repeats each. An increase in protein levels can be seen in both Wt (increase of 11%) and the PC2 level in *Pkd2*$^{lrm4/lrm4}$ (increase of 45%) samples. The absolute increase in normalised protein level on MG132 treatment remains similar between the two genotypes. The levels in *Pkd2*$^{lrm4/lrm4}$ cells was not however, restored to the Wt.

Supplementary references.

1 GARCIA-GONZALEZ, M. A., OUTEDA, P., ZHOU, Q., ZHOU, F., MENEZES, L. F., QIAN, F., HUSO, D. L., GERMINO, G. G., PIONTEK, K. B. & WATNICK, T. 2010. Pkd1 and Pkd2 are required for normal placental development. *PLoS One,* 5.
