# Supplementary material for: Ciliary exclusion of Polycystin-2 promotes kidney cystogenesis in an autosomal dominant polycystic kidney disease model
Source: Nat Commun. 2019 Sep 6;10:4072. doi: 10.1038/s41467-019-12067-y (PMC6731238; doi:10.1038/s41467-019-12067-y)
Supplement: Supplementary file 3 — Reporting Summary [file 41467_2019_12067_MOESM3_ESM.pdf]

nature research

Corresponding author(s): Norris and Walker

Last updated by author(s): Jul 22, 2019

Reporting Summary

Nature Research wishes to improve the reproducibility of the work that we publish. This form provides structure for consistency and transparency in reporting. For further information on Nature Research policies, see Authors & Referees and the Editorial Policy Checklist.

Statistics

For all statistical analyses, confirm that the following items are present in the figure legend, table legend, main text, or Methods section.

n/a	Confirmed	
☐	☒	The exact sample size (n) for each experimental group/condition, given as a discrete number and unit of measurement
☐	☒	A statement on whether measurements were taken from distinct samples or whether the same sample was measured repeatedly
☐	☒	The statistical test(s) used AND whether they are one- or two-sided<br>*Only common tests should be described solely by name; describe more complex techniques in the Methods section.*
☒	☐	A description of all covariates tested
☒	☐	A description of any assumptions or corrections, such as tests of normality and adjustment for multiple comparisons
☐	☒	A full description of the statistical parameters including central tendency (e.g. means) or other basic estimates (e.g. regression coefficient) AND variation (e.g. standard deviation) or associated estimates of uncertainty (e.g. confidence intervals)
☒	☐	For null hypothesis testing, the test statistic (e.g. F, t, r) with confidence intervals, effect sizes, degrees of freedom and P value noted<br>*Give P values as exact values whenever suitable.*
☒	☐	For Bayesian analysis, information on the choice of priors and Markov chain Monte Carlo settings
☒	☐	For hierarchical and complex designs, identification of the appropriate level for tests and full reporting of outcomes
☒	☐	Estimates of effect sizes (e.g. Cohen's d, Pearson's r), indicating how they were calculated

Our web collection on statistics for biologists contains articles on many of the points above.

Software and code

Policy information about availability of computer code

Data collection	no software used
Data analysis	N glycosylation sites were predicted usingNetNGlyc1.0 in silico glycosylation site prediction software, as noted in our methods, referenced (ref #70) and accessoible online : http://www.cbs.dtu.dk/services/NetNGlyc/

For manuscripts utilizing custom algorithms or software that are central to the research but not yet described in published literature, software must be made available to editors/reviewers. We strongly encourage code deposition in a community repository (e.g. GitHub). See the Nature Research guidelines for submitting code & software for further information.

Data

Policy information about availability of data

All manuscripts must include a data availability statement. This statement should provide the following information, where applicable:

- Accession codes, unique identifiers, or web links for publicly available datasets
- A list of figures that have associated raw data
- A description of any restrictions on data availability

Provide your data availability statement here.

Field-specific reporting

Please select the one below that is the best fit for your research. If you are not sure, read the appropriate sections before making your selection.

☒ Life sciences ☐ Behavioural & social sciences ☐ Ecological, evolutionary & environmental sciences

Life sciences study design

All studies must disclose on these points even when the disclosure is negative.

Sample size	Embryonic kidneys: the mutant phenotype is fully penetrant, all lrm4 homozygous embryos exhibit oedema etc. Therefore, the kidney cyst phenotype was considered to also be fully penetrant (indeed, it was present on all homozygous lrm4 embryos that we analysed). Kidneys were selected from 5 embryos from independent litters to be processed for histology.<br>Biochemistry: pools of embryonic kidneys were assessed to find a pool size which gave a reliable and detectable amount of protein. 8 pools of three pairs of kidneys, per genotype were used to reduce variation and provide sufficient sample for one technical repeat.
Data exclusions	no data exclusions
Replication	all attempts at replication were successful
Randomization	IF imaging and SIM: Each cell within a field was selected randomly for image acquisition. Cilia were selected at random before PC2 staining was assessed in order to avoid collecting a biased representation of data.
Blinding	Blinding was used whenever possible. Image analysis was performed on genotype blinded files.

Reporting for specific materials, systems and methods

We require information from authors about some types of materials, experimental systems and methods used in many studies. Here, indicate whether each material, system or method listed is relevant to your study. If you are not sure if a list item applies to your research, read the appropriate section before selecting a response.

Materials & experimental systems

n/a	Involved in the study	
☐	☒	Antibodies
☐	☒	Eukaryotic cell lines
☒	☐	Palaeontology
☐	☒	Animals and other organisms
☒	☐	Human research participants
☒	☐	Clinical data

Methods

n/a	Involved in the study	
☒	☐	ChIP-seq
☒	☐	Flow cytometry
☒	☐	MRI-based neuroimaging

Antibodies

Antibodies used	Mouse anti-acetylated tubulin (working concentration 2 µg/ml, Sigma-Aldrich, T7451)<br>mouse anti-ARL13B (working concentration 2 µg/ml, Abcam, ab136648)<br>mouse anti-CEP164 (working concentration 5 µg/ml, Sigma, SAB2702133)<br>mouse anti-PC1 (working concentration 1 µg/ml for IF, 0.4 µg/ml for WB, 7E12, Santa Cruz, SC130554)<br>rabbit anti-PC2 (working concentration 1 µg/ml for both IF and WB , H-280, Santa Cruz, SC25749)<br>mouse anti-β-actin (working concentration 0.1 µg/ml for WB, Sigma, A5316)<br>mouse anti-Tubulin (working concentration 0.1 µg/ml for WB, Sigma, T9026).<br>goat-anti-IFT88 (working concentration 1 µg/ml, Abcam, ab42497)<br>mouse-anti-Gamma-tubulin (working concentration 1 µg/ml, Sigma, T6557)
Validation	Western blot internal control antibodies Tubulin (Sigma) and β-actin (Sigma) are routinely used in our lab and have been validated using a variety of mammalian cell lines and tissues. A specific band at the expected molecular weight is observed with low background noise to signal ratio.<br><br>PC1-7e12 (Santa Cruz) and PC2-H280 (Santa Cruz): Validation for western by tagged recombinant protein expression from full-length cDNA expression constructs in mammalian cells. These were used as positive controls. Protein null tissues or cells were used to control for the specific detection of any protein products. Thus providing negative controls. The correct identification of proteins in the positive samples was demonstrated by the lack of the signals in null samples.<br><br>Arl13b (Abcam)and IFT88 (Abcam) are routinely used in our lab for IF. The Abcam website states that these antibodies have been validated for western blot, IF and IHC.<br>Cep164 (Sigma) and gamma tubulin (Sigma) are also routeinly used in our lab. The Sigma website states that these antibodies have been validated and are suitable for IF, IHC, WB and IP.

Eukaryotic cell lines

Policy information about cell lines

Cell line source(s)	primary cell lines made by our lab from mutant mouse lines- Mouse Embryonic Fibroblasts (MEFs)
Authentication	PC2 expression was assessed in PC2-lrm4 MEFs and compared with PC2-/- and Wt MEFs.
Mycoplasma contamination	Cells were not tested for mycoplasma due to being a primary cell line. Cells were maintained at a low passage.
Commonly misidentified lines (See ICLAC register)	N/A

Animals and other organisms

Policy information about studies involving animals; ARRIVE guidelines recommended for reporting animal research

Laboratory animals	Mouse, C57BL/6J, pregnant females were used to provide embryos of both sexes for analysis. Embryos were assessed between E14.5 and E15.5. MEFs were made from embryos between E13.5 and E14.5
Wild animals	Study did not involve wild animals
Field-collected samples	Study did not involve field collected animals
Ethics oversight	All experiments were performed under the guidelines and approval of the MRC Harwell Ethics Committee and the UK Home Office

Note that full information on the approval of the study protocol must also be provided in the manuscript.
